# Multi-contrast submillimetric 3 Tesla hippocampal subfield segmentation protocol and dataset

**DOI:** 10.1038/sdata.2015.59

**Published:** 2015-11-10

**Authors:** Jessie Kulaga-Yoskovitz, Boris C. Bernhardt, Seok-Jun Hong, Tommaso Mansi, Kevin E. Liang, Andre J.W. van der Kouwe, Jonathan Smallwood, Andrea Bernasconi, Neda Bernasconi

**Affiliations:** 1 Neuroimaging of Epilepsy Laboratory, Department of Neurology and Neurosurgery and McConnell Brain Imaging Centre, McGill University, Montreal, Quebec, Canada H3A2B4; 2 Max-Planck Institute for Human Cognitive and Brain Sciences, Department of Social Neuroscience, Leipzig 04303, Germany; 3 Medical Imaging Technologies, Healthcare Technology Center, Siemens Medical Solution USA, Inc., Princeton, New Jersey 08540, USA; 4 Department of Radiology, Athinoula A. Martinos Center for Biomedical Imaging, Massachusetts General Hospital, Charlestown, Massachusetts 02129, USA; 5 Department of Psychology, York University, York YO105DD, UK

**Keywords:** Magnetic resonance imaging, Brain imaging, Brain, Neurology, Neuroscience

## Abstract

The hippocampus is composed of distinct anatomical subregions that participate in multiple cognitive processes and are differentially affected in prevalent neurological and psychiatric conditions. Advances in high-field MRI allow for the non-invasive identification of hippocampal substructure. These approaches, however, demand time-consuming manual segmentation that relies heavily on anatomical expertise. Here, we share manual labels and associated high-resolution MRI data (MNI-HISUB25; submillimetric T1- and T2-weighted images, detailed sequence information, and stereotaxic probabilistic anatomical maps) based on 25 healthy subjects. Data were acquired on a widely available 3 Tesla MRI system using a 32 phased-array head coil. The protocol divided the hippocampal formation into three subregions: subicular complex, merged Cornu Ammonis 1, 2 and 3 (CA1-3) subfields, and CA4-dentate gyrus (CA4-DG). Segmentation was guided by consistent intensity and morphology characteristics of the densely myelinated molecular layer together with few geometry-based boundaries flexible to overall mesiotemporal anatomy, and achieved excellent intra-/inter-rater reliability (Dice index ≥90/87%). The dataset can inform neuroimaging assessments of the mesiotemporal lobe and help to develop segmentation algorithms relevant for basic and clinical neurosciences.

## Background & Summary

The hippocampus has been a focus of neuroscience research for decades. Highly segregated connectional properties have promoted its use as a model system. The hippocampus plays an important role in multiple cognitive processes, particularly declarative memory^1,2^; its structural compromise is a hallmark of prevalent neurological and psychiatric disorders, such as temporal lobe epilepsy^3^, Alzheimer’s disease^4,5^, depression^6^, and schizophrenia^7^.

Prior to the advent of sophisticated histological staining techniques^8^, the hippocampal formation was described as a single entity despite its complex histo-morphology. Since the description by Ramon y Cajal^9^, several histological subdivisions have been proposed^10–12^. Similarly, neuroimaging studies have generally considered the hippocampus as a single structure, constrained by limited spatial resolution^13^. Developments in high-field MRI at 3 Tesla and beyond, together with the use of phased-array head coils, offer new opportunities to appraise its internal structure by unveiling strata rich in white matter, and improved identification of the hippocampal sulcus, which separates Cornu Ammonis (CA) and subiculum from the dentate gyrus (DG). Paralleling advances in hardware, a number of studies have provided MRI-based guidelines to manually segment hippocampal subfields^14–23^. While substantial progress has been made, challenges remain, particularly when attempting to separate individual CA subfields from one another, which compromises reliability within and across analysts. From a practical perspective, manual segmentations require anatomical expertise and are often prohibitively time-consuming.

Here, we share a dataset containing manual segmentations of hippocampal subfields together with submillimetric multi-spectral images in 25 healthy individuals. To facilitate local implementation and independent verification, we share detailed MR sequence information as well; importantly, all data were acquired in a clinically-feasible scan time on a widely available 3 Tesla MRI system.

Opting for high reliability, segmentations were based on a protocol that divided the hippocampal formation into consistently identifiable subregions, guided by intensity and morphology of the densely myelinated molecular layer, together with few geometry-based boundaries flexible to overall mesiotemporal anatomy. Specifically, we combined presubiculum, parasubiculum, and subiculum proper into a single label (subiculum), joined CA1, 2, and 3 (CA1-3), and merged CA4 with the DG (CA4-DG). While segmentation relied primarily on T1-weighted (T1w) data, T2-weighted (T2w) images offered additional guidance. We provide the full set of multispectral images in high-resolution native and stereotaxic (MNI152) space, the manual labels, together with a probabilistic atlas that can inform functional and structural imaging assessments of the hippocampal formation. Moreover, our datasets can be used to develop new protocols, validate existing ones and design automated algorithms relevant for basic as well as clinical neurosciences.

## Methods

### Participants

We studied 25 healthy individuals (12 males; 21–53 years, mean±s.d. age=31.2±7.5 years; Table 1), recruited through advertisement. All participants had normal or corrected-to-normal vision; none of them suffered from neurological, psychiatric, or somatic diseases. The Ethics Committee of the Montreal Neurological Institute and Hospital approved the study and written informed consent was obtained from all participants in accordance with the standards of the Declaration of Helsinki. Participants gave their written informed consent prior to scanning and received a monetary compensation.

### Scan parameters

MRI data were acquired on a 3 Tesla Siemens TimTrio scanner using a 32-channel head coil. We obtained two sets of T1w images: a 3D magnetization-prepared rapid-acquisition gradient echo (MPRAGE) with millimetric resolution (repetition time (TR)=2,300 ms; echo time (TE)=2.98 ms; inversion time (TI)=900 ms; flip angle=9°; matrix size=256×256; field-of-view (FOV)=256×256 mm^2^; 176 sagittal slices with 1 mm slice thickness resulting in 1×1×1 mm^3^ voxels; iPAT=2, acquisition time=5.30 min), and a submillimetric 3D MPRAGE (TR=3,000 ms; TE=4.32 ms; TI=1,500 ms; flip angle=7°; matrix size=336×384; FOV=201×229 mm^2^; 240 axial slices with 0.6 mm slice thickness resulting in 0.6×0.6×0.6 mm^3^ voxels; acquisition time=16.48 min; to increase the signal-to-noise ratio, two identical scans were acquired, motion corrected, and averaged into a single volume). T2w images were obtained using a 2D turbo spin-echo sequence (TR=10,810 ms; TE=81 ms; flip angle=119°; matrix size=512×512; FOV=203×203 mm^2^, 60 coronal slices angled perpendicular to the hippocampal long axis, slice thickness=2 mm, resulting in 0.4×0.4×2.0 mm^3^ voxels; acquisition time=5.47 min).

### Pre-processing

**Figure 1:**
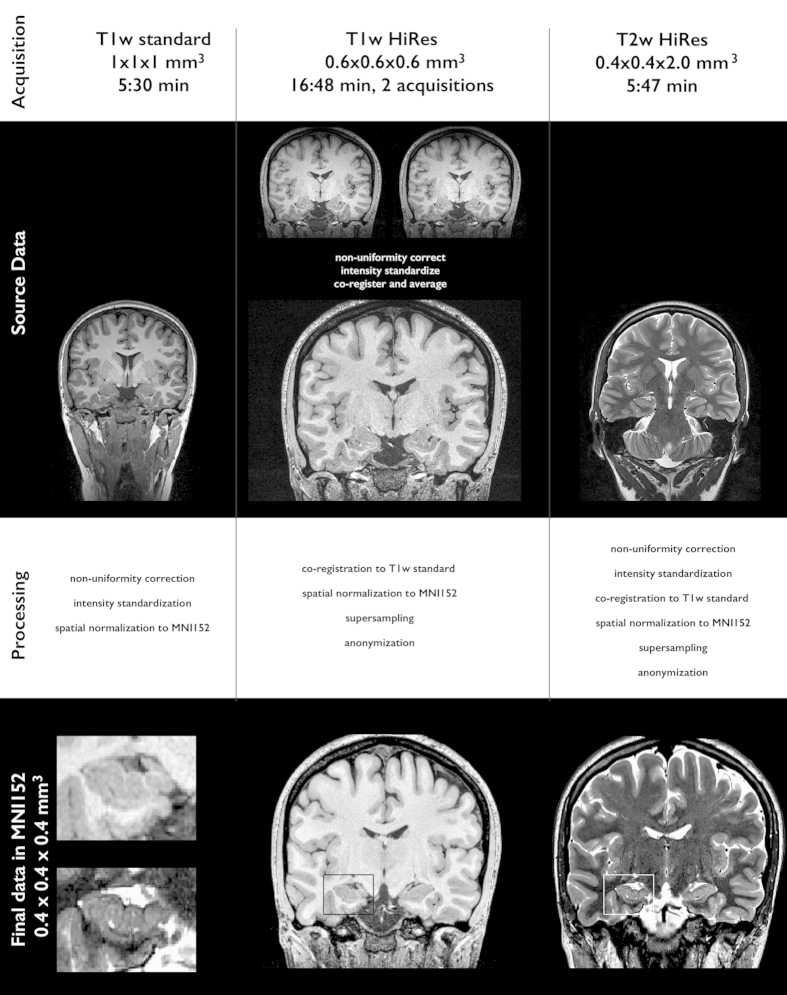
Dataset: schematic illustration of image acquisition, native data, image processing and final processed data.

MRI data files were converted from DICOM to MINC (*.mnc) format using dcm2mnc with dicom header anonymization. Images underwent automated correction for intensity non-uniformity and intensity standardization^24^. Millimetric and submillimetric T1w MRI volumes were linearly registered to the high-resolution MNI-ICBM152 template^25,26^. T2w images were linearly registered to the millimetric T1w MRI in native space; the resulting transformation matrix was concatenated with the matrix that mapped the millimetric T1w image to the MNI space, thereby linearly mapping T2w images to this template. During the final registration of submillimetric T1w and T2w data to MNI space, images were resampled to a resolution of 0.4×0.4×0.4 mm^3^, yielding a voxel volume of 0.064 mm^3^. To reduce interpolation artifacts given the anisotropic resolution of the T2w data, we applied a non-local up-sampling method that recovers high frequency information using a data-adaptive patch-based reconstruction together with a subsampling coherence constraint^27^. MNI-space structural scans were subsequently anonymized by zeroing out the voxels in the vicinity of the facial surface, teeth, and auricles following a previously described procedure^28^. For data sharing, images were converted to NIfTI (*.nii) format using mnc2nii. Please see Fig. 1 for a schematic overview of the preprocessing steps and data quality.

### Protocol description

**Figure 2:**
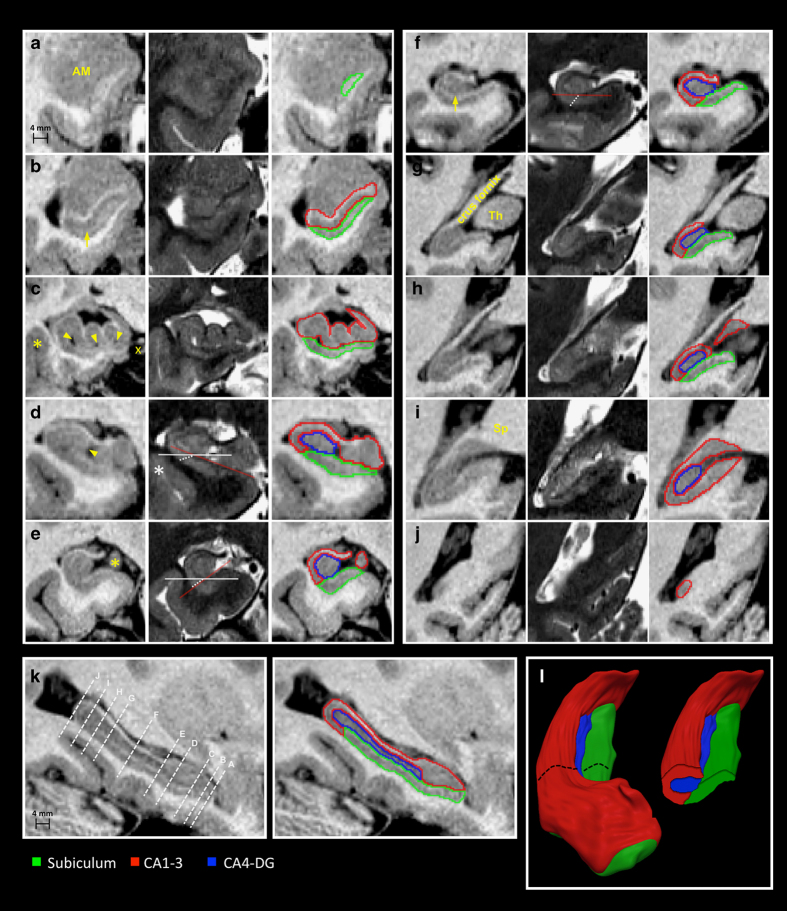
Anatomical boundaries of hippocampal subfileds on T1- and T2-weighted MRI. Sections displaying critical landmarks are shown. (**a**,**j**) are the most rostral and caudal coronal sections. (**a**) The rostral-most tip of the hippocampus is composed of the subiculum; at this level, the alveus surrounds the subiculum, separating it from the overlying amygdala (AM). (**b**) When CA1 first becomes visible, it runs parallel to the subiculum; the structures are separated by the subicular molecular layer (arrow). (**c**) Vertical digitations of CA1-3 (arrowheads point to cavities within the hippocampal sulcus; x indicates the ambient cistern); the supero-lateral subicular interface with CA1 is drawn along a line following the hippocampal sulcus, directed towards the fundus of the collateral sulcus (asterisk). (**d**) The rostral-most portion of CA4-DG is set at the section where the medial portion of the DG, the margo denticulatus, becomes visible (arrowhead). (**e**) Junction between head and body, at the level of the uncal apex (asterisk). (**f**) Hippocampal body; the arrow points to the molecular layer of the subiculum. (**g**) Rostral portion of hippocampal tail: the crus fornix is fully visible and well demarcated from the thalamus (Th). (**h**) The caudal slice of the subiculum is set to the posterior-most section on which the thalamus can be identified. (**i**) Middle segment of the tail. The subiculum is replaced by CA1-3, at the level at which the crus fornix fuses with the splenium (Sp) of the corpus callosum. (**j**) Terminal segment of the tail. (**k)** Sagittal hippocampal section displaying planes of the coronal cuts. (**l**) 3D surface rendering of hippocampal subfields with a coronal cut at the level of the body. On coronal sections, the orientation of the hippocampal body varies across individuals, modifying the spatial relationships between subiculum and CA1. In **d**, the hippocampus is oriented clockwise. In **e**, it is oriented counter-clockwise and in **f** it has a horizontal position. The red line follows the slope of the superior border of the subiculum, the solid white line represents the horizontal axis, and the dashed white line is placed at the boundary between subiculum and CA1.

A single rater (JKY), blinded to case identities, carried out all segmentations using a 3D viewer (http://www.bic.mni.mcgill.ca/ServicesSoftwareVisualization/). Subfield segmentation took approximately 16 h per individual (8 h per hemisphere). Boundaries were based on anatomical descriptions of the hippocampus by Duvernoy^29^ and Insausti^30^. As spatial relationships between subfields vary along the hippocampal long axis, landmarks are separately described for the hippocampal head (Fig. 2a–e), body (Fig. 2f), and tail (Fig. 2g–j). These segments were defined as in our previous protocol^31^.

Segmentations were primarily performed on coronal T1w images, with cross-referencing to sagittal/axial views. T2w data eased the identification of the densely myelinated and thick molecular layer of the subiculum (forming its superior border). This layer is hyperintense on T1w and hypointense on T2w images (Fig. 2b–i, k); it is contiguous with, but distinct from the thinner molecular layer of CA1 (ref. 30). The second landmark is the molecular layer of the DG and that of CA fused across the vestigial hippocampal sulcus; this ensemble is visible as a T1w-hyperintense/T2w-hypointense band (Fig. 2c–i). The molecular layers, along with residual vascular cavities that follow the sulcal route, consistently appear on T2w images and separate the DG from the subiculum (inferiorly and medially) and the CA (inferiorly, laterally, and superiorly). We included alveus and fimbria in the CA1-3 label.

#### a) Hippocampal head

The hippocampal head includes the subiculum, CA1-3, and small portions of the DG. Its rostral-most section is composed of the subiculum only^30^ (Fig. 2a). Here, the alveus surrounds the subiculum, separating it from the overlying amygdala; cross-referencing to the sagittal view confirmed this boundary. The inferior subicular boundary is formed by parahippocampal white matter running along the entire rostro-caudal extent of the hippocampus. Perforant projections from the entorhinal cortex to the subiculum occasionally blurred this boundary; in this case, we identified the subiculum by cross-referencing to axial/sagittal views. As the exact boundary between subiculum and infero-medial entorhinal cortex cannot be visualized on MRI, it was defined by extending a straight line along the gray-white matter border at the crown of the parahippocampal gyrus until it reached the cerebro-spinal fluid in the ambient cistern^32^.

When CA1 first becomes visible, it runs parallel to the subiculum; for a few slices, the molecular layer of the subiculum separates both structures, with CA1-3 on the top (Fig. 2b). More posteriorly, given the overlap (rather than sharp transition) between the pyramidal layers of CA1 and subiculum^30^, we drew a line along the hippocampal sulcus pointing towards the fundus of the collateral sulcus (Fig. 2b,c). This often-oblique line has been previously used to describe this boundary^19^.

The hippocampal head exhibits 3–4 digitations before turning medially to form the posterior uncus. Each digitation encapsulates an extension of the DG. At the level of the head, however, the DG molecular layer that would have allowed for its identification cannot be visualized. For consistency, we merged CA and DG at this level (Fig. 2c). We could reliably segment CA4-DG at the junction of head and body, where the medial surface of the DG (known as margo denticulatus) becomes visible (Fig. 2d).

#### b) Hippocampal body

**Figure 3:**
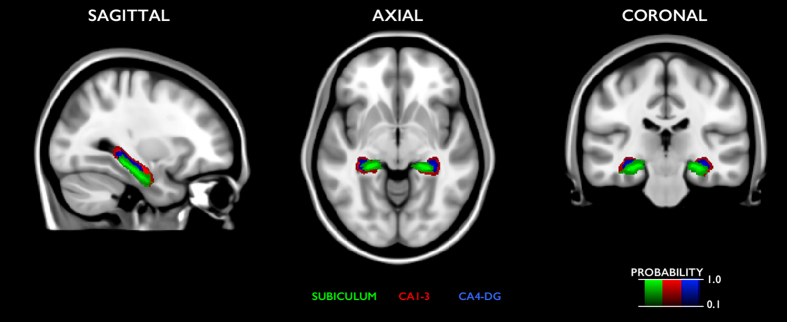
Statistical probabilistic atlas of hippocampal subfields overlaid on the MNI152 template.

Head and body interface at the caudal end of the uncus^31^ (Fig. 2e,f). Here, the margo denticulatus of the DG has a characteristic toothed appearance and is separated from the overhanging fimbria by the fimbriodentate sulcus. Coronally, the orientation of the hippocampal body varies along its rostro-caudal direction both across and within individuals. The term malrotation has been coined to describe this abnormal shifting/rotations of the long hippocampal axis relative to the horizontal plane^33,34^, which likely affects the relative boundary between subiculum and CA1. To determine this border, we adapted our guidelines based on the position of the hippocampus on coronal slices: (1) if the left hippocampus was oriented counter-clockwise (clockwise for the right hippocampus), the boundary was defined as the extension of the line corresponding to the superior subicular border (Fig. 2e); (2) if the hippocampus was horizontally positioned, the border was defined as a line drawn from the lateral-most point of the subicular molecular layer at a 45 degrees angle until it reached the underlying white matter (Fig. 2f); (3) if the left hippocampus was oriented clockwise (counter-clockwise for the right), the border followed a line drawn from the lateral-most point of the subicular molecular layer towards the fundus of the collateral sulcus (Fig. 2d). Inferior and medial boundaries of the subiculum remained the same as in the head.

CA and DG form two U-shaped interlocking laminae, one fitting into the other, and separated from each other by the hippocampal sulcus. For consistency, voxels corresponding to the fused molecular layers of the CA1-3 and DG were assigned to CA4-DG. As the CA3-CA4 boundary cannot be resolved on MRI, the superior border of CA4-DG was drawn as the horizontal continuation of the hippocampal sulcus, from its most medially visible point towards the fimbriodentate sulcus.

#### c) Hippocampal tail

The junction between body and tail was set as the rostral-most slice at which the crus fornix becomes fully visible (Fig. 2g)^31^. In the initial segment of the tail, the CA1-subiculum boundary was determined to be the infero-lateral extension of the superior subicular border (Fig. 2g,h). Inferior and medial borders of the subiculum were defined as in the body. In the initial portion of the tail, CA1 is deeply located, hidden by the subiculum; more posteriorly, it appears at the surface of the parahippocampal gyrus, progressively replacing the subiculum. The exact posterior subicular border is not visible on MRI: we consistently chose it to be the posterior-most coronal slice on which the thalamus could be seen (Fig. 2h,i), verified on sagittal view. We excluded the isthmus of the cingulate gyrus, which replaces the subiculum in the middle and terminal segments of the tail, by excluding grey matter inferior to the hippocampal sulcus, best visualized sagittally. The hippocampal sulcus separates the DG from the subiculum in the initial segment, and from CA1-3 in the initial and middle segments. Furthermore, the fused molecular layers of CA and DG allowed us to visualize the caudal border of the DG on the sagittal view.

The posterior hippocampal end belongs to CA1-3 (Fig. 2j) and faces the cerebrospinal fluid of the lateral ventricle medially and of the atrium laterally. This boundary was best seen sagittaly (Fig. 2k). While fimbria and alveus were included in CA1-3, we excluded the crus fornix (Fig. 2g). The latter joins the splenium of the corpus callosum.

### Code availability

All MRI preprocessing employed standard routines (non-uniformity correction, intensity normalization, image registration). We used minc tools that are freely available on github (https://github.com/BIC-MNI/minc-tools). Similar processing can also be achieved using tools provided by other freely available packages, such as FreeSurfer (http://freesurfer.net) or FSL (http://fsl.fmrib.ox.ac.uk/). The patch-based up-sampling technique for T2w-images is available on P. Coupé’s website (https://sites.google.com/site/pierrickcoupe/softwares/super-resolution-for-3d-mri/monomodal). Defacing was based on publically available code (https://surfer.nmr.mgh.harvard.edu/fswiki/mri_deface).

## Data Records

The submillimetric 3 Tesla dataset are highly suitable for the development and cross-validation of future manual or automatic segmentation protocols. MRI data and subfield segmentations of all participants, detailed scan parameters, as well as stereotaxic probabilistic maps are available on Dryad (Data Citation 1) and NITRC (Data Citation 2). A README file with a detailed description of the content of all downloads is available there as well. MRI data files were converted from to DICOM to MINC format (using dcm2mnc) before processing, and to NIfTI (using mnc2nii) after processing. For every subject, high-resolution T1w and T2w data are available in 0.4 mm isotropic MNI152 space as well as in their native spaces. For registration purposes, the 1×1×1 mm^3^ T1w data is also provided in native and stereotaxic space. Labels in NIfTI format of the subiculum, CA1-3 and CA4-DG are provided in the high-resolution MNI152 space. We furthermore provide probabilistic anatomical maps of each subfield in 1×1×1 mm^3^ MNI152 space. To anonymize data, centre-specific study and participant codes have been removed using an automated procedure. MRI data have been de-faced. All participants were given sequential integer IDs with an ‘S’ prefix.

## Technical Validation

### Contrast-to-noise ratio

To obtain a quantitative index of MRI data quality, we estimated Contrast-to-Noise ratio (CNR), similar to the approach carried out in a recently published study^21^. In short, an eroded mask of the CA1-3 was compared with an equivalently-sized mask of the temporal lobe white matter inferior to it. The CNR was estimated using the following formula:

$$CNR=\frac{\overset{¯}{WM}-\overset{¯}{GM}}{\sqrt{var\left(WM\right)+var\left(GM\right)}}$$

where $\overset{¯}{WM}$ and $\overset{¯}{GM}$ are the mean intensities in the WM and GM masks; *var(.)* is the intensity variance. We calculated the CNR for each subject in native T1w and T2w space, as well as in the MNI space on which segmentations were performed. For native T1w and T2w data, mean±s.d. (range) CNR estimates across the sample were: 3.04±0.23 (2.73–3.48) and 4.42±0.64 (3.29–5.83). For supersampled and MNI space data, corresponding values were 4.74±0.86 (3.27–7.73) and 4.53±0.59 (3.63–5.71). Please see Table 2 for a subject-by-subject listing.

### Intra- and inter-rater reliability

JKY segmented subfields of 10 hippocampi (5 left, 5 right) from 10 different subjects twice, 6 months apart. We assessed inter-rater reliability comparing subfield delineations of 10 hippocampi segmented by JKY and another observer (KL), blinded to each other’s segmentation. Reliability was quantified using Dice overlap indices between two labels^35^, *D=2×|M*_*1*_ *∩M*_*2*_ *|/(|M*_*1*_ *|+|M*_*2*_ *|)×100%*, where *M*_*1*_ is the 1st label, *M*_*2*_ the 2nd label; *M*_*1*_ *∩ M*_*2*_ is the intersection of *M*_*1*_ and *M*_2_; *|.|* is the volume operator. We also calculated intra-class correlations (ICC). The Dice index quantifies the overlap of two labels geometrically, whereas ICC calculates statistical similarity. To approximate the actual distribution of reliability values, we employed 1,000 bootstrap-based subsamplings and computed 95% confidence intervals.

Table 3 displays mean±s.d. as well as bootstrap confidence interval of Dice indices for individual subfileds. Overall, indices were ≥90 and 87% for intra- and inter-rater reliability, respectively. The ICC ranged from 0.91 to 0.96 within and from 0.73 to 0.91 between raters.

### Probabilistic anatomical maps

For each MNI152-space subfield label, we generated statistical anatomical maps that outline the probability of subfield location across participants (Fig. 3).

## Usage Notes

The procedures we employed in this study resulted in a high-resolution 3 Tesla dataset containing submillimetric MRI data in native and MNI152 space, together with manual labels of three hippocampal subfields in MNI152 space. Data are shared in documented standard formats, such as NIfTI or plain text files, to enable further processing in arbitrary analysis environments with no imposed dependencies on proprietary tools. Exam card printouts from the scanner are also available for local implementation of the image acquisition protocol. All processing performed on the released data article were produced by openly accessible software on standard computer workstations. Data are available on a curated open access repository (Data Citation 1) and on NIRTC (Data Citation 2).

## Additional Information

**How to cite this article:** Kulaga-Yoskovitz, J. *et al.* Multi-contrast submillimetric 3 Tesla hippocampal subfield segmentation protocol and dataset. *Sci. Data* 2:150059 doi: 10.1038/sdata.2014.59 (2015).

## Supplementary Material



## Figures and Tables

**Table 1:** Samples, subjects and data outputs.

**Subjects**	**Age**	**Gender**
S01	24	Female
S02	21	Female
S03	26	Male
S04	30	Male
S05	22	Female
S06	30	Female
S07	31	Female
S08	40	Male
S09	28	Female
S10	29	Male
S11	32	Male
S12	26	Female
S13	29	Male
S14	46	Male
S15	27	Female
S16	27	Male
S17	27	Female
S18	43	Female
S19	29	Male
S20	30	Female
S21	53	Female
S22	29	Female
S23	28	Male
S24	38	Male
S25	34	Male

**Table 2:** Contrast-to-noise estimates.

**Subject**	**T1w MNI**	**T2w MNI**	**T1w native**	**T2w native**
S01	7.23	4.76	3.48	4.95
S02	3.85	5.08	3.14	4.18
S03	3.27	3.90	2.97	4.36
S04	5.23	4.46	3.16	4.21
S05	4.28	4.82	3.26	4.66
S06	5.34	5.36	3.14	4.73
S07	5.03	4.07	3.14	4.02
S08	4.71	4.49	3.12	4.49
S09	5.46	4.61	3.40	3.97
S10	4.37	3.80	2.74	3.56
S11	4.47	5.01	2.75	4.49
S12	5.84	4.56	3.12	4.86
S13	4.15	4.18	2.78	3.68
S14	5.44	3.63	2.97	3.29
S15	5.55	3.86	3.16	4.00
S16	4.86	4.58	2.83	5.50
S17	3.69	5.30	3.15	4.91
S18	4.86	4.44	3.18	4.25
S19	3.89	4.07	2.83	4.24
S20	5.54	3.71	3.00	3.52
S21	4.17	4.21	2.75	4.97
S22	4.84	5.62	3.43	5.83
S23	4.72	4.20	2.83	4.01
S24	3.84	4.82	2.89	4.39
S25	3.84	5.71	2.73	5.48

**Table 3:** Intra-and inter-rater reliability assessment.

	**Subiculum**	**CA1-3**	**CA4-DG**	**Whole hippocampus**
Intra-rater				
Dice (%)	90.5±1.6 (89.5–91.3)	92.9±1.0 (92.3–93.4)	90.0±1.9 (89.0–90.9)	92.6±0.6 (95.9–96.8)
ICC	0.94	0.91	0.96	0.92

inter-rater				
Dice (%)	87.1±5.3 (84.3–89.2)	90.3±3.6 (88.4–91.7)	87.6±4.8 (85.0–89.3)	94.3±2.2 (93.1–95.2)
ICC	0.73	0.91	0.90	0.95

## References

[d1] Dryad Kulaga-Yoskovitz 2015 http://dx.doi.org/10.5061/dryad.gc72v

[d2] NITRC Kulaga-Yoskovitz 2015 http://www.nitrc.org/projects/mni-hisub25

